# Two-point Compression Ultrasound Technique Risks Missing Isolated Femoral Vein DVTs

**DOI:** 10.5811/westjem.2022.2.53830

**Published:** 2022-06-03

**Authors:** Matthew Tabbut, Nate Ebersole, Lauren Icken, Robert Jones, Diane Gramer

**Affiliations:** * MetroHealth Medical Center, Department of Emergency Medicine, Cleveland, Ohio; † MetroHealth Medical Center, Department of Medicine/Pediatrics, Cleveland, Ohio; ‡ Albany Medical Center, Department of Emergency Medicine, Albany, New York

## Abstract

**Background:**

Deep vein thrombosis (DVT) is a common vascular problem seen in the emergency department (ED) and is commonly identified using ultrasound performed by a vascular lab, the radiology department, or at the point of care. Previous studies have assessed the utility of a two-point vs sequential technique to identify the presence of a thrombus. One particular study reported a concerning rate of isolated femoral vein thrombi that would be missed by a two-point technique.

**Objectives:**

In this study we sought to determine whether the two-point technique misses isolated femoral vein thrombi.

**Methods:**

We conducted a retrospective review of patients who had a new diagnosis of DVT in the ED diagnosed with vascular lab, radiology, or point-of-care ultrasound to assess for the presence and rate of thrombi that would be missed using a two-point scanning technique.

**Results:**

We included in our study 356 patients with a diagnosis of new DVT. In our population, 21 (5.9%; 0.95 confidence interval: 3.7%, 8.9%) patients were identified with thrombi isolated to the femoral vein.

**Conclusion:**

The two-point technique for lower extremity vascular ultrasound is insufficient for ruling out proximal DVTs in ED patients.

## INTRODUCTION

Deep venous thrombosis (DVT) is a common vascular problem seen in the emergency department (ED) with implications for patient morbidity or mortality if untreated.^1^ Traditionally, duplex ultra-sound involving compression and Doppler techniques has been used as the safest and most effective method of identifying the presence or absence of DVT.^2^ Point-of care-ultrasound (POCUS) performed by physicians at the bedside, focusing on two-dimensional (2D) compression, has been shown to be a safe and effective method of diagnosing proximal lower extremity DVTs in the ED.^3^ Since publication of early literature supporting this practice, the use of POCUS for this application has grown. The American College of Emergency Physicians has included compression ultrasound in the list of core applications of bedside ultrasound that emergency physicians are able to perform.^4^

Current guidelines for duplex ultrasonography of the lower extremity recommend sequential visualization of the deep venous system with compression of the proximal greater saphenous vein, common femoral vein, femoral vein, and popliteal vein.^5^ However, several studies suggest that an abbreviated two-point technique focusing on branch points around the greater saphenous junction and bifurcation of the common femoral vein around the groin and popliteal trifurcation around the knee may be as effective as sequential compression in evaluating for DVT.^2^,^6^ In fact, one meta-analysis indicates that the two-point technique is equivalent to sequential compression,^7^ making this an attractive approach in a fast-paced environment such as the ED.

This abbreviated method of scanning, however, has raised concern for missing focal DVTs that do not extend through one of the two planes scanned using the two-point technique. Adhikari and colleagues reported a concerning number of thrombi that would be missed by the two-point technique.^8^ In this study we sought to validate the findings of Adhikari et al to determine whether the two-point technique is insufficient to identify the presence of an isolated femoral vein thrombus.

## METHODS

We conducted a single-center, retrospective study of patients presenting to the ED between 2010–2015 who had received imaging for initial diagnosis of suspected lower extremity DVT. The time period was chosen to replicate the study conditions of Adhikari et al. We performed our study in a large, urban, academic ED with an annual volume of approximately 90,000 patients with an established point-of-care ultrasound program, emergency medicine residency, and emergency ultrasound fellowship. This study was approved by the institutional review board.

We identified patients based on an *International Classification of Diseases* (ICD) query of the electronic health record. Adult patients ages ≥18 years were included if they had received imaging for suspected lower extremity DVT and were diagnosed in the ED with an acute lower extremity DVT. We also included patients identified in outpatient clinics and sent to the ED for same-day initial management of DVT. Acute lower extremity DVT was defined as a thrombus at or proximal to the popliteal vein and had not been reasonably previously identified.

The patients must have received an ultrasound either from the institution’s dedicated vascular lab (images interpreted by vascular surgery), radiology department (images interpreted by radiologists), or at the bedside by trained emergency physicians or the emergency ultrasound team using standard department protocols. The standard protocol for ED-performed ultrasound in our department includes imaging the greater saphenous/common femoral vein junction, bifurcation of the common femoral vein, three locations on the femoral vein (proximal, mid, and distal), and the popliteal vein using the sequential compression technique. Both the radiology department and vascular lab use the sequential compression approach with color and spectral Doppler when evaluating the lower extremity vasculature. Calf vein findings were variably reported; thus, we did not include patients with isolated calf thrombi in our study. For the purposes of this study, the two-point technique is defined as compression ultrasound of the greater saphenous/common femoral junction region and the popliteal vein region, excluding evaluation of the common femoral vein.

*Population Health Research Capsule*

What do we already know about this issue?

*Deep vein thrombosis (DVT) is commonly identified in the emergency department using ultrasound.*

What was the research question?

*Could we validate previous findings that the two-point technique misses an unacceptable rate of thrombi isolated in the femoral vein in a similar patient cohort?*

What was the major finding of the study?

*The two-point technique for lower extremity DVT evaluation missed 5.9% of thrombi isolated to the femoral vein.*

How does this improve population health?

*Understanding the test characteristics of bedside ultrasound protocols helps to improve patient care and decrease rates of morbidity and mortality.*

The patients identified by ICD query were reviewed by participating medical students for inclusion and exclusion criteria. The ICD query searched for all patients diagnosed in the ED with an acute DVT. Patients were excluded from final analysis if the DVT was not of the lower extremity, if it was chronic or previously known, or the DVT had been diagnosed later in the patient’s hospital course (not in the ED). For those patients included in the final analysis, we reviewed the imaging reports for the location of the clot in the lower extremity.

We recorded the presence of thrombus at the distal external iliac vein, greater saphenous vein \, common femoral vein (, proximal femoral vein, mid femoral vein, distal femoral vein, and popliteal vein. Uncertain findings were adjudicated by three members of the study team (MT, DG and RJ). Findings were recorded on a deidentified Excel spreadsheet (Microsoft Corp., Redmond, WA) spreadsheet for data analysis. We calculated confidence intervals (CI) using an online calculator found at https://sample-size.net/confidence-interval-proportion.

## RESULTS

The initial ICD query resulted in 1,493 patient events. After review of the health records we excluded 1,137 patients who did not meet the inclusion criteria for the following reasons: no DVT was identified; the DVT had been diagnosed later in the hospital course; or the patient had chronic DVT, non-lower extremity DVT, or previously known DVT. A total of 356 patients met inclusion criteria and were included in the final analysis. The mean age of the included patients was 53 with a standard deviation of 15.

The proportion of studies performed by the ED ultrasound team, the radiology department, and the vascular lab are shown in Table 1. Of the 356 with an acute isolated lower extremity DVT, most were found to extend across more than one section of the lower extremity as shown in Table 2. The most common location was a thrombus extending from the proximal femoral vein through the popliteal vein. Isolated thrombi were found in each of the vein segments of interest. The rates of isolated thrombi in each of the vein segments are shown in Table 3. The most common location for an isolated thrombus was in the popliteal vein (18%, 0.95 CI: 14.1%, 22.4%) followed by the femoral vein (5.9%, 0.95 CI: 3.7%, 8.9%).

## DISCUSSION

The use of two-point vs sequential technique when evaluating for DVT has been a point of discussion for POCUS users with proponents arguing that DVTs most commonly occur at branch points, extend through multiple segments, or would be otherwise recognized based on sonographic factors other than compression (ie, alterations in Doppler flow). Given the importance of the findings of Adhikari et al on POCUS DVT ultrasound workflow, we sought to validate previous findings that identified a significant rate of isolated thrombi in areas that would be missed when strictly using the two-point technique for DVT evaluation. In 2014, Adhikari and colleagues conducted a retrospective review of patients seen in their ED over a five-year period who had a comprehensive ultrasound of the lower extremity and had been diagnosed with a DVT. In their analysis they identified 2451 patients who had undergone duplex ultrasound evaluation with DVTs identified in 362 of those patients. Of those 362 patients, 20 (5.5%) had thrombi isolated to the femoral vein and three (0.8%) had thrombi isolated to the deep femoral vein.

The utility and safety of the two-point technique was then called into question, leading to dis-cussion on the most efficient and appropriate way to conduct this exam at the bedside.^8^ The vast majority of patients in our study had thrombi that traversed through multiple zones (popliteal, femoral, common femoral, etc). Specifically, we noted that the rate of isolated femoral vein thrombus and isolated deep femoral vein thrombus was similar to that found by Adhikari. Our data on the number of isolated DVTs that could be missed by the two-point technique highlights the external validity of their observations.

Previous literature has shown that direct visualization of a patent vessel as demonstrated by com-pression in a sequential analysis is a reliable way of evaluating for DVT in the ED.^2^,^9^,^10^ Doppler is often used in radiology departments and vascular labs, in addition to direct compression to assess for filling defects and direct or augmented flow alterations due to thrombi located outside the area of direct visualization. Demonstration of a filling defect can be easily over- or under-demonstrated with inappropriate Doppler settings. Doppler flow alterations, which are predicated on the presumption that a thrombus is occlusive, have not been shown to identify thrombi that were not previously visualized with compression ultrasound.^11^ Thus, quality sequential 2D compression ultrasonography is vitally important, especially for non-occlusive thrombi.

Based on our data, we believe that strict adherence to the two-point technique is insufficient to adequately evaluate the proximal vessels of the lower extremity for DVT. We believe that POCUS sonographers should perform sequential compression of the proximal leg veins that includes the femoral vein.

## LIMITATIONS

Our study does have several limitations. While we sought to replicate the methods of the Adhikari study (including a similar five-year window), there were several differences that need to be acknowledged when interpreting our results. First, this was a single-center study with ultrasounds performed by emergency physicians, the radiology department, or the vascular lab in an effort to ensure capture of as many patients as possible who had been diagnosed with DVT. Thus, there were several formats of reporting that required interpretation or confirmation by the study team. Questions regarding reporting language discrepancy were confirmed by a registered vascular technologist sonographer dedicated to the ED or by an attending physician trained and privileged in bedside ultrasound.

Second, this analysis was a retrospective study in which patients were found by searching through a database by ICD codes. Thus, patient identification was dependent on proper input of ICD codes by coders based on ED diagnosis. Patients improperly coded would thereby not be identified. However, we did include in our analysis patients who were originally missed but identified by ICD on subse-quent visits to maintain reasonable accuracy in patient inclusion.

Finally, like the comparison study that we sought to validate, we conducted a retrospective analysis of ultrasound studies using the sequential compression technique. We visualized the location of the thrombi in the various portions of the proximal leg and extrapolated that the presence of an isolated thrombus in the femoral vein would result in a missed DVT using the two-point compression technique. While this is likely true when performing a limited DVT ultrasound in the ED using compression as the method to identify thrombi, it does not account for flow alterations that may be visualized with Doppler proximal to the site of a thrombus. Since routine use of Doppler is not typically performed in ED ultrasound, this was not assessed in our study. For these reasons, further analysis in a prospective manner would be warranted.

## CONCLUSION

Our results demonstrate that the two-point technique for lower extremity vascular ultrasound is insufficient for ruling out proximal DVTs in ED patients. A prospective analysis of two-point vs sequential compression would be warranted to confirm these findings.

## Figures and Tables

**Table 1:** Proportion of studies performed in each department to identify deep vein thromboses.

Performing department	Percentage of studies (N)
ED POCUS	12.4% (44)
Radiology	39.3% (140)
Vascular Lab	48.3% (172)

*ED*, emergency department; *POCUS*, point-of-care ultrasound.

**Table 2:** Number and rate of lower extremity thrombi spanning multiple regions of the deep leg veins.

Thrombus location	Percentage (N)	95% Confidence interval
DEI-POP	15.5% (55)	11.9 – 19.6%
DEI-FVd	3.4% (12)	1.8 – 5.8%
CFV-POP	12.6% (45)	9.4 – 16.6%
CFV-FVd	3.7% (13)	2.0 – 6.2%
FVp-POP	28.1% (100)	23.5 – 33.1%

*DEI*, distal external iliac; *CFV*, common femoral vein; *FVp*, proximal femoral vein; *FVd*, distal femoral vein; *POP*, popliteal vein.

**Table 3:** Number and rate of isolated lower extremity thrombi.

Isolated thrombus location	Percentage (N)	95% Confidence interval
DEI	0.6% (2)	0.7 – 2.0%
CFV	2.3% (8)	1.0 – 4.4%
GSV	2.5% (9)	1.2 – 4.7%
DFV	0.6% (2)	0.7 – 2.0%
FV (P/M/D)	5.9% (21)	3.7 – 8.9%
POP	18.0% (64)	14.1 – 22.4%

*DEI*, distal external iliac; *CFV*, common femoral vein; *GSV*, greater saphenous vein; *DFV*, deep femoral vein; *FV*, proximal femoral vein (proximal, mid or distal); *POP*, popliteal vein.
